# Expression Characterization of Six Genes Possibly Involved in Gonad Development for Stellate Sturgeon Individuals (*Acipenser stellatus*, Pallas 1771)

**DOI:** 10.1155/2018/7835637

**Published:** 2018-03-29

**Authors:** Alexandru Burcea, Gina-Oana Popa, Iulia Elena Florescu (Gune), Marilena Maereanu, Andreea Dudu, Sergiu Emil Georgescu, Marieta Costache

**Affiliations:** ^1^Department of Biochemistry and Molecular Biology, Faculty of Biology, University of Bucharest, Bucharest 050095, Romania; ^2^S.C. Danube Research-Consulting S.R.L., Isaccea 825200, Romania

## Abstract

Nowadays, in sturgeon's aquaculture, there is a necessity for sex identification at early stages in order to increase the efficiency of this commercial activity. The basis for a correct identification is studying the different factors that influence the gonad development. The research has been directed towards molecular methods that have been employed with various degrees of success in identifying genes with different expression patterns between male and female sturgeons during their development stages. For the purpose of understanding the sexual development of 4-year-old stellate sturgeon *(Acipenser stellatus)* individuals, we have selected six genes (*foxl2*, *cyp17a1*, *ar*, *dmrt1*, *sox9*, and *star*). We analysed the gene expression of the selected genes for gonads, anal fin, liver, body kidney, and white muscle. The *cyp17a1*, *ar*, *dmrt1*, and *sox9* genes have a significant higher expression in male gonads than in female gonads, while the data shows no significant differences in the expression of the investigated genes in the other organs. We investigate these genes to shed light on aquaculture sturgeon sexual development.

## 1. Introduction

Sturgeons and paddlefish form the order Acipenseriformes, which is a group of fish that have existed since at least the Lower Jurassic and nowadays are threatened in their entire range of distribution [1].

Caviar is harvested from sturgeons and it has been on high demand, even though they are threatened species, making them economically valuable in world trade [2]. The sevruga caviar is harvested from *A. stellatus* females and is one of the highest-priced caviar types. For this type of caviar to be harvested naturally, the females must reach maturity, which is at 8–10 years. In contrast, males reach maturity at 5-6 years [3, 4]. There is also a period of more than one year between subsequent spawning [5, 6].

Even though aquaculture can provide a supply of sturgeon-derived products, such as caviar and meat, and can also help in conservation efforts providing offspring for repopulation [7], the early sex identification of individuals could further help the aquaculture efforts through correctly separating the males from more economically profitable females. More affordable sturgeon products could lower the pressure of poaching, sustaining the *ex situ* conservation efforts. Also, aquaculture could benefit from the knowledge of genetic architecture and sex determination in sturgeons [8]. For distinguishing females from males, a good understanding of the processes of sexual development is needed. Therefore, studies of the possible sex-determining mechanisms are important for identifying a starting point.

In nature, there are different types of conserved patterns of sex-determining mechanisms which range from male heterogamety (XY) found in the majority of mammals and female heterogamety (ZW) found in most birds to environmental sex determination (ESD) expressed most commonly as temperature sex determination (TSD) found in reptiles and some fish species [9]. Fish species have complex patterns of sex determination which range from hermaphroditism, with individuals presenting both female and male gonads, and gonochorism, where each individual presents only female or male gonads, to TSD. There is also the possibility of natural gynogenesis, where the female ovule is penetrated by a sperm cell, but the sperm nucleus does not contribute to embryo formation which in turn inherits only the female chromosomes [10]. Genetic sex determination was described in *Oryzias latipes* which has an early type of XY genetic system [11]. Cytogenetic studies of fish chromosomes showed that the karyotype can be employed in a low number of fish species for identifying the sex of the individual [12]; furthermore, in the case of sturgeons, this method did not identify any sex-specific chromosomes [13].

The number of fish species that present male heterogamety compared to female heterogamety is higher [12]. One hypothesis is that the XY and ZW systems originated in ancestral species with environmental sex determination in which either the male or the female had the size advantage as an adult; thus, the XY system is present in species where fitness correlates with male size, and the ZW system is found in species where females have the size advantage [14]. It has also been observed that the system of sex determination may depend on reproduction advantage [15]. Wild *Acipenser transmontanus* individuals have a 1 : 1 sex ratio between males and females [16] which means that the ratio is not influenced by the environment. This creates the hypothesis that the system of sex determination in sturgeons is genetic and not environmental, which is backed up by gynogenesis experiments suggesting sex ratios in conformity with the ZW system where gynogenesis may produce ZZ males, WW superfemales, and ZW females. This is the case of *Acipenser baerii* [17], *Acipenser brevirostrum* [18], *Acipenser nudiventris* [19], *A. transmontanus* [20], Bester hybrid (*Huso* female × *Acipenser ruthenus* male) [21], and *Polyodon spathula* [22] even though there is no direct evidence from karyotype morphology [23].

Sturgeons do not present sexual dimorphism that could help in the early selection of females from less economically valuable males [13]; therefore, an extensive plethora of methods have been utilized with the objective of identifying the sex of sturgeon individuals, such as biopsy for *H. huso* [24, 25]; endoscopy for *Acipenser gueldenstaedtii* [26], *H. huso* [26], and *Acipenser oxyrinchus* [27]; and ultrasonography for *A. stellatus* [6]. Even though these methods have high accuracy, the lowest age at which the sex of an individual could be identified is between 1 and 3 years through invasive methods or ultrasonography. Also, noninvasive molecular methods, such as random amplified polymorphic DNA (RAPD) for *A. baerii*, *Acipenser naccarii*, *A. ruthenus* [28], *H. huso* [29], and *Acipenser fulvescens* [30]; amplified fragment length polymorphism (AFLP) for *A. baerii* and *A. gueldenstaedtii* [28]; and intersimple sequence repeats (ISSR) for *A. naccarii*, *A. baerii*, and *A. gueldenstaedtii* [28], have been utilized in order to see if any DNA fragments are specific to females or males, because of the high possibility that the sex-determining system is genetic. These methods did not provide any results that could discern between females and males. Because of this, the studies have shifted towards next-generation sequencing (NGS) studies. In the case of sturgeons, which have complex genomes, NGS allows the transcriptome analysis and gives information on possible differences between males and females in expression and presence of various genes (*dmrt1*, *tra-1*, *wt1*, *lhx1*, *cyp19A1*, *fhl3*, *fem1a*, *gsdf*, *foxl2, ar*, *emx2*, *cyp17a1*, etc.) that may be involved in the sexual development processes in different sturgeon species [7, 31–33]. With NGS laying the groundwork for gene discovery, the next step was the analysis of gene pathways using qPCR studies; therefore, the most-studied genes in vertebrates that are involved in male pathway were *sox9*, *amh*, *nr5a1*, *nrob1*, and *wt1* [34–36]. In the case of sturgeons, there have been a series of studies regarding genes involved in male or female sex development: Sertoli cell factors *dmrt1* and *sox9*, Leydig cell factors *star* and *cyp17a1*, and other genes such as *lh*, *ar*, *vtg*, *foxl2*, *fsh*, and *igf1* [35–41], showing that the genes are expressed differently in the various tissues investigated. Even if there are studies that investigate sturgeon sex development, the underlying mechanisms are still poorly understood, and no master sex gene has been identified as of yet.

In this study, we investigated the expression of six genes that are known from literature to be steroid-related Leydig cell factors (*cyp17a1*, *ar*, and *star*), male gonad genes that are Sertoli cell factors (*dmrt1* and *sox9*), and a female gene for ovarian differentiation (*foxl2*). These genes were chosen to investigate if they are involved in the sexual development of specific tissues in *A. stellatus*. Different organs were analysed alongside gonads in order to showcase if the studied genes are specific to gonad development. This study aims at observing the expression pattern between males and females and between the different organs (gonads, white muscle, body kidney, anal fin, and liver) in order to establish if these genes are involved in the sexual development of *A. stellatus* and if they are gonad specific. This is the first time that the expression of these genes was investigated through qPCR for this species.

## 2. Materials and Methods

### 2.1. Samples

Four female and four male *A. stellatus* 4-year-old individuals from aquaculture, kept in ponds with water recirculation and temperature of 22°C, were anesthetized with clove oil : ethanol (1 : 10) in water; their branchial arches were sectioned, and they died of blood loss. After dissection, the individuals were sexed by observing the presence of lamellae on the female gonads and the relatively smooth male gonads as in Flynn and Benfey [42]. At the age of 4 years, the individuals were at early maturation stage, undergoing gametogenesis. Females had previtellogenic oocytes, while males presented primary spermatocytes. The liver, white muscle, body kidney, gonads, and anal fin fragments were collected in RNAlater Stabilization Reagent (Qiagen).

### 2.2. RNA Isolation

The isolation of total RNA was performed from 10 mg of tissue using the PureLink RNA Mini Kit (Thermo Fisher) for the liver, body kidney, and gonads, while the RNeasy Fibrous Tissue Mini Kit (Qiagen) was used for the 20 mg of white muscle and anal fin following the manufacturer's protocol. After isolation, 10 *μ*L of RNA solution was digested with DNase I (Qiagen) for removal of contaminant genomic DNA using 10 *μ*L RDD buffer, 2.5 *μ*L DNase I, and 77.5 *μ*L RNase-free ultrapure water in a 100 *μ*L final volume. The quantity and purity of total RNA samples were determined using the NanoDrop 8000 (Thermo Scientific). RIN values of the RNA samples were determined using Agilent RNA 6000 Nano Kit (Agilent) and Agilent 2100 Bioanalyzer using the manufacturer's protocol. Samples with RIN values smaller than 8 were not included in further analysis.

### 2.3. Reverse Transcription

For cDNA synthesis, 1000 ng of total RNA was reverse transcribed using the iScript Reverse Transcription Supermix for RT-qPCR (BioRad) following the manufacturer's protocol.

### 2.4. Primer Design

Because of the lack of data regarding genes involved in the sexual development of *A. stellatus*, GenBank sequences from other sturgeon species were used to design pairs of primers (Table 1). The pairs of primers were designed in silico using Primer BLAST NCBI, tested for annealing temperature using the Primer3 online software, and tested for specificity by using BLAST NCBI. The *foxl2*, *cyp17a1*, *ar*, *dmrt1*, *sox9*, *star*, *β*-*actin*, *gapdh*, and *28S rRNA* amplicons were sequenced using the primers in Table 1 on the ABI Prism 3130 Genetic Analyzer (Applied Biosystems), using the BigDye Terminator v3.1 Cycle Sequencing Kit (Applied Biosystems), and the cDNA sequences were deposited in GenBank with the following accession numbers: KX420678-KX420686 (Table 1).

### 2.5. qPCR

The qPCR was carried out on the iCycler iQ Real-Time PCR Detection System (BioRad) using the iQ SYBR Green Supermix Kit (BioRad) in 25 *μ*L final volume with 400 nM of each primer from Table 1, 100 ng of cDNA, 12.5 *μ*L of iQ SYBR Green Supermix 2X, and 10.5 *μ*L of DNase-free ultrapure water. The incubation program was comprised of initial denaturation (95°C for 5 minutes), cycling stages (35 cycles of 95°C denaturation for 30 seconds, 58°C annealing for 30 seconds, and 72°C extension for 30 seconds with data collection after each extension), and a melt curve stage (from 55°C to 97.5°C with an increment of 0.5°C for 10 seconds and data collection at each increment). The raw data points were represented by the quantification cycle (*C*_q_) as stated in “The MIQE Guidelines: Minimum Information for Publication of Quantitative Real-Time PCR Experiments” [43]. The analysis was performed in triplicate for each gene, and the validity of the qPCR was confirmed by analysing the melting curves. The amplification efficiency (*E*) of the investigated genes was situated between 94% and 105%, while the linear standard curve (*r*^2^) was higher than 0.994 for all genes.

### 2.6. Data Analysis

The reference genes' (*gapdh*, *β-actin*, and *28S rRNA*) *C*_q_ results for each group were tested for stability using NormFinder 20 [44]. The best single result was observed for *β-actin* (0.088), while the best result for a combination of genes was for the arithmetic mean of *gapdh* and *β-actin* (0.061), the latter combination being used in the subsequent analysis because of the good score.

The relative expression value (2^−Δ*C*q^) was obtained by normalization, subtracting the arithmetic mean of the *β-actin* and *gapdh* reference genes from each gene of interest [45]. The normal distribution of the dataset groups was tested using the Shapiro-Wilk test implemented in IBM SPSS 19.0 [46, 47]. We used the one-way ANOVA test (*p* ≤ 0.05) with Tukey correction for multiple comparisons implemented in GraphPad Prism 6.01 in order to investigate the difference in gene expression between 4-year-old *A. stellatus* males and females.

## 3. Results

The *β*-*actin* and *gapdh* arithmetic mean was chosen to normalize the data from the six genes of interest. The expression levels for the genes of interest are presented in Table 2 in the form of means and ±SD, along with one-way ANOVA results for the comparison between 4-year-old *A. stellatus* males and females.

**Figure 1:**
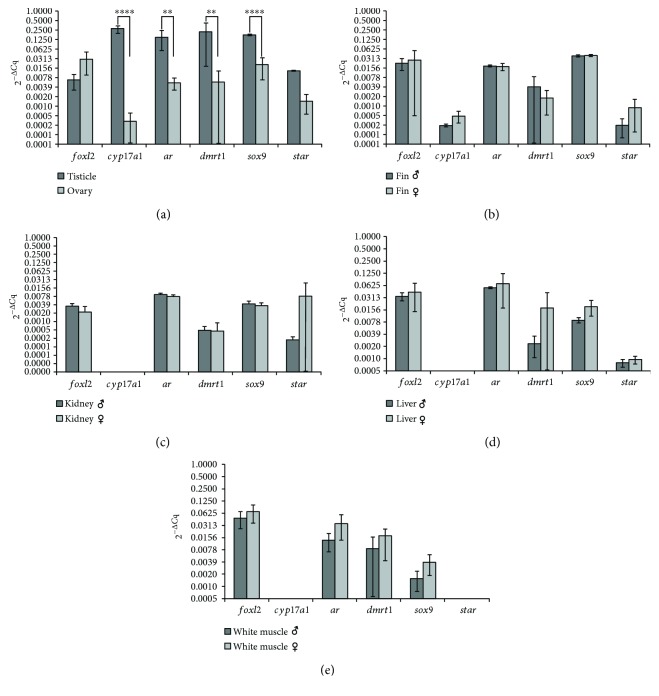
The 2^−Δ*C*q^ comparison of *foxl2*, *cyp17a1*, *ar*, *dmrt1*, *sox9*, and *star* genes between males and females in the gonads (a), anal fin (b), body kidney (c), liver (d), and white muscle (e). The arithmetic mean of *gapdh* and *β-actin* reference genes was used for normalization. The data points are represented by arithmetic means ± SD on a logarithmic scale in base two. The statistical significance of the difference in expression was tested with one-way ANOVA using Tukey correction (^∗∗^*p* ≤ 0.01 and ^∗∗∗∗^*p* ≤ 0.0001).

A statistically significant difference (*p* ≤ 0.01) between females and males was observed only in gonads for the *cyp17a1*, *ar*, *dmrt1*, and *sox9* genes, for which the males presented higher levels of expression than the females (Figure 1(a)). In the case of body kidney, white muscle, and liver, the expression of *cyp17a1* was not observed. The expression of *cyp17a1* was observed only in gonads and anal fin (Figures 1(a) and 1(b)). The expression of the *star* gene was not detected for white muscle, but it was observed in the rest of the investigated organs (gonads, anal fin, body kidney, and liver) (Figure 1). The *foxl2* and *star* genes did not present any statistically significant difference in expression between the sexes for this organ. The difference in expression between females and males for the investigated genes was not statistically significant for the rest of the organs (anal fin, body kidney, liver, and white muscle) (Figures 1(b)–1(e)).

**Figure 2:**
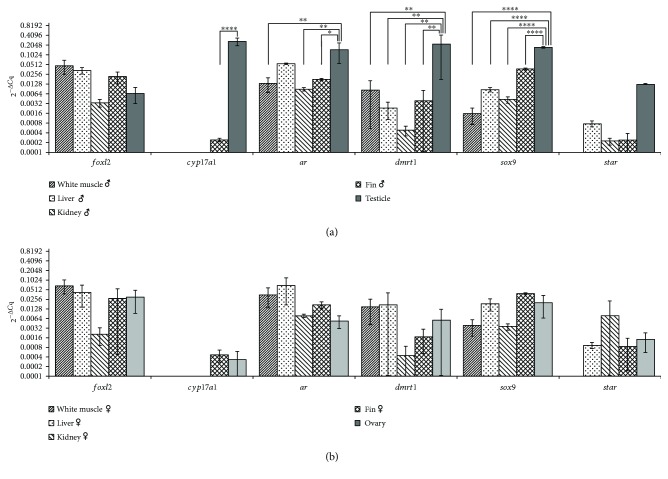
The 2^−Δ*C*q^ comparison of *foxl2*, *cyp17a1*, *ar*, *dmrt1*, *sox9*, and *star* genes between organs from males (a) and females (b). The arithmetic mean of *gapdh* and *β-actin* reference genes was used for normalization. The data points are represented by arithmetic means ± SD on a logarithmic scale in base two. The statistical significance of the expression comparison was tested with one-way ANOVA using Tukey correction (^∗^*p* ≤ 0.05, ^∗∗^*p* ≤ 0.01, and ^∗∗∗∗^*p* ≤ 0.0001).

For males, a difference in the expression (*p* ≤ 0.0001) for the *cyp17a1* gene between the gonad and anal fin was observed, with higher levels in the gonad. For the *ar* gene, the expression in the male gonad was statistically different from that in the anal fin (*p* ≤ 0.05), in the body kidney (*p* ≤ 0.01), and in the white muscle (*p* ≤ 0.01), the highest levels being found in the gonad but not different from that in liver (Figure 2(a)). Higher levels of *dmrt1* gene were found in the testicle than in the anal fin, body kidney, liver, and white muscle (*p* ≤ 0.01). The *sox9* levels were statistically higher (*p* ≤ 0.0001) in the gonads than in the anal fin, body kidney, liver, and white muscle (Figure 2(a)). For the *foxl2* and *star* genes, no difference in expression between the testicle and the other organs was observed. For females, no statistically significant difference in expression between the gonads and the other organs was observed (Figure 2(b)).

## 4. Discussion

There could be a difference in expression between males and females for the reference genes that could be explained by the cease in expression that occurs during spermiogenesis because of chromatin construction or transcription factors that inhibit the transcription of certain genes [38, 48, 49]. Therefore, the statistically significant difference in expression between males and females, regarding the *cyp17a1*, *ar*, *dmrt1*, and *sox9* genes could be due to a difference in expression of the reference genes. But because of prior observed differences reported on various sturgeon species using NGS techniques [7, 31–33] alongside our approach of using two reference genes to try and limit this possibility of different expression of reference genes, we consider this a good method for determining the true state of expression of the investigated genes.

It is very likely that the determination and consequent differentiation of sturgeon gonads take place during the first year of life, studies showing gonad surface differentiation at 4 months for *A. baerii*, at 4 months for *A. gueldenstaedtii*, at 180 days for *A. naccarii*, at 6 months for *A. brevirostrum*, at 8 months for *A. ruthenus*, and at 6 months for the Bester hybrid [50]. There are no studies regarding the onset of gonadal differentiation in *A. stellatus* individuals, but because the majority of sturgeons present first year differentiation of gonads, it could be feasible that *A. stellatus* also presents this characteristic. At 4 years old, the individuals have undergone the onset of differentiation and are in the primary spermatocyte stage, in the case of males (spermatogenesis), and previtellogenic oocytes, in the case of females (oogenesis).

There is no evidence for a master sex-determining gene present only for males or females, observed through NGS in the case of *A. fulvescens* studies, even though there is evidence of differential gene expression between males and females [7, 31]. On the other hand, *dmrt3*, *igf-1*, *lhx1*, and *sox11* genes were found to be specific to the testicle transcriptome, while *cyp19A1a*, *foxl2*, *gnrhr*, and *nanos3b* were only found in the ovary transcriptome of *Acipenser sinensis* [33]. For *A. naccarii*, five genes were found to be specific for male (*wt1*, *lhx1*, *cyp19A1*, *fhl3*, and *fem1a*) and two (*ar*, *emx2*) for female libraries [32]. Contrary to these studies, we have found an expression of *foxl*2 and *ar* in all organs tested from both females and males which rules them out as master sex-determining genes in *A. stellatus* individuals. Because of the fact that all the genes we investigated were present both in males and females, we consider that they are not master sex-determining genes for *A. stellatus* individuals.

For the system of sex determination, the lack of *dmrt1* expression in females is consistent with an XY system which appears not to be the case in sturgeons [40], where both females and males present the expression of this gene, which is in pattern with the ZW system of sex determination, where in birds, *dmrt1* plays the role of master sex-determining gene [9]. It could be feasible that the *dmrt1* gene is expressed at higher levels in sturgeon males, independent of the number of copies that are present in the genome due to double dosage which appears for ZZ/ZW species. This has been observed in our study and in other research involving sturgeon species, such as *A. fulvescens* 13-14-year-old individuals (adolescent female and mature male) [31], *A. baerii* 3-year-old individuals with immature gonads [36, 39], *A. baerii* 16–18-month-old juveniles [35, 39], *A. gueldenstaedtii* 550-day-old and 1600-day-old individuals [40], and *A. sinensis* 3- and 4-year-old individuals. This could be due to specific targeting of *dmrt1* gene by an unknown factor that determines a higher expression in males. It could also be feasible that in females, there are factors that modulate the expression of *dmrt1* so that it is lower than in males. One such factor could be a microRNA molecule that specifically targets the *dmrt1* gene in females, silencing or reducing its expression as also observed in silkworm where a piRNA molecule can inhibit the *masc* gene leading to the formation of female individuals [51].

There is a lack of sexual dimorphism regarding the *dmrt1* gene in the case of *A. gueldenstaedtii* 9-month-old juveniles with undifferentiated gonads [7], *A. sinensis* 3-year-old gametogenetic juveniles [33], and *Scaphirhynchus platorynchus* above 2-year-old individuals with fully developed gonads [38], which could be due to the developmental stage at which the gonads were sampled. The lack of expression of the *dmrt1* gene could be due to incomplete coverage in NGS or the developmental stage at which the 6-month-old *A. naccarii* individuals were at the time of sampling [32]. The different developmental stages and species that were investigated show a nonspecific time of expression of the *dmrt1* gene in the case of sturgeons. Each species may have a specific moment in the development of the gonads at which the expression of *dmrt1* occurs in different mRNA levels between females and males. The *dmrt1* gene, a candidate for double expression in males, is mostly expressed in higher levels in males than in females, but there are developmental stages of sturgeons in which the *dmrt1* gene is not expressed in different levels [7, 33, 38]. Even though this pattern of expression could be species specific, it could be argued that the expression of *dmrt1* starts at the onset of differentiation and decreases to a stable level after the gonads are fully developed. This is why no difference in expression could be distinguished for *A. gueldenstaedtii* 9-month-old individuals with undifferentiated gonads [7] and for adult *S. platorynchus* individuals with fully developed gonads [38]. The fact that for *A. stellatus* male individuals the expression of *dmrt1* is higher than in females shows that this gene is involved in the development of the male gonads at the spermatogenesis stage.

The *sox9* gene has been found in *Acipenser sturio* and *A. fulvescens* in the genome of both males and females, through sequencing [31, 52], and is overexpressed in the testicles when compared to ovaries in immature 3-year-old *A. baerii* individuals [36, 39]. We observed a higher expression of *sox9* in male gonads that might indicate that the gene is involved in the development of testicles. In the case of 16-month-old juvenile *A. baerii* individuals with differentiated gonads, the expression of *sox9* in gonads is not different between females and males [35, 39], the same as in *A. gueldenstaedtii* 9-month-old individuals which indicates that the *sox9* gene is not involved in the onset of differentiation of sturgeon gonads. In *A. sinensis* 3-year-old individuals, the gene was present in both females and males through NGS [33], similar to 6-month-old *A. naccarii* individuals regarding the same gene [32]. For the *A. stellatus* individuals, the expression of *sox9* at 4 years old shows that this gene is involved in gametogenesis.

The *foxl2* gene has a higher expression in females for *S. platorynchus* individuals [38] and for *A. gueldenstaedtii* 9-month-old juvenile presumptive females [7]. This is in contrast to our observation that the difference in expression is not statistically significant between males and females. The *foxl2* gene is involved in the onset of female gonad differentiation [7], and in the case of *S. platorynchus* [38], it appears to be involved in female gonad differentiation because of the age of the sturgeons investigated. These results may be due to the different species studied; in our case, the individuals were well past starting differentiation and well into gonadal stages like spermatogenesis and oogenesis; therefore, we rule out the possibility of *foxl2* involvement in development at this stage for *A. stellatus* even though it is in contrast to what was previously described for *S. platorynchus*.

The *cyp17a1* gene is known to be a Leydig cell factor modulating androgen production, and a higher expression in male gonads than in female gonads is to be expected. We have observed this higher expression between males and females, and we also observed that it is not expressed in other tissues except for anal fin. The expression in the anal fin is lower than that of male gonads but not different from that of female gonads. In immature *A. baerii* gonads (16-month-old and 3-year-old individuals), the expression of *cyp17a1* was higher in males; however, different from our discovery is that, in this case, the expression was also detected in the muscle, liver, and kidney [39], which was not observed in our study. This pattern of expression, higher in male gonads than in female gonads, could be due to the need for steroidogenic hormones in males during gametogenesis for which the *cyp17a1* gene is responsible. The expression of the *cyp17a1* gene, which is involved in converting progestins into androgens in testicles, is correlated with the expression of the *ar* gene, which encodes for an androgen receptor. Both being in higher levels in males than in females in the case of *A. baerii* and in our own study, this coupled with the age at which this pattern is observed could indicate that androgens are the main mediators of vertebrate masculinization [39, 53, 54] and in this case sturgeon male gonad development, after testicular differentiation especially for *A. stellatus* of 4 years of age where the lamellae are clearly visible for female gonads and the surface differentiation already took place. The *cyp17a1*, *ar*, *dmrt1*, and *sox9* genes have different expression patterns in male gonads than in female gonads, which could indicate that these are probably involved in the development of male gonads. The *foxl2* gene is involved in female gonad differentiation in other species, but no significant difference in expression was detected so that we can conclude the same thing for the stage at which the *A. stellatus* individuals were when sampling took place. The *star* gene is normally involved in the male developmental pathway; however, the individuals we investigated may be over the age at which the difference in expression occurs.

Besides the *cyp17a1* and *ar* genes that are possibly involved in the male gonad development pathway, we also observed the expression of the *star* gene. The expression of the *star* gene in our study proved it was not significantly different between females and males, which is in contrast to the situation of *A. baerii* individuals [39]. The *star* gene is involved in steroid synthesis and should have a higher expression after gonadal differentiation. For our samples, this is correlated with reaching gonadal maturity at four years which is near the five-year mark for *A. stellatus* male maturity [3, 4].

## 5. Conclusions

We have observed that *cyp17a1*, *ar*, *dmrt1*, and *sox9* genes are involved in the gonad development of *A. stellatus* because of the significant difference in expression between males and females. The fact that the *cyp17a1*, *ar*, *dmrt1*, and *sox9* genes have a higher expression in male gonads than in the other organs tested could imply that these genes are involved in the male pathway for gonad development.

No significant difference in the expression of the investigated genes was detected in tissues that could be used for noninvasive sex identification in early stages (white muscle and anal fin).

Pinpointing the developmental stage at which these genes start presenting a difference in expression could help in the study of sexual development in sturgeons; the data presented here could be the groundwork for future studies that will focus on younger individuals of the same species. Our results are in agreement with other sturgeon research that has attempted to find a master sex-determining gene which would suggest that it has yet to be discovered.

## Figures and Tables

**Table 1:** Primers for qPCR analysis.

GenBank	Gene	PCR product length (bp)	Primer name	Primer sequence
KX420683	*ar*—*androgen receptor*	197	ar-F	5′-CKTGACTCCCCGAACAATCA-3′
			ar-R	5′-AAGGTAGCACGCTGGAACTC-3′

KX420684	*dmrt1*—*doublesex and mab-3 related transcription factor 1*	129	dmrt1-F	5′-CCACCCTGTTCCACTTCCAG-3′
			dmrt1-R	5′-GAAGWGGATGGTGCTGTGCT-3′

KX420685	*sox9*—*sex-determining region Y-box 9*	115	sox9-F	5′-AGGCCGATTCCYCTCACTCT-3′
			sox9-R	5′-TGCAYGTCTGTTTTGGGAGT-3′

KX420686	*foxl2*—*forkhead box L2*	120	foxl2-F	5′-GCCCACCTCGTACAATCCTT-3′
			foxl2-R	5′-CTTAGCTGCTGAGGGTGGTG-3′

KX420678	*cyp17a1*—*cytochrome P450 family 17 subfamily A polypeptide 1*	134	cyp17a1-F	5′-CCGTCGCTTACCTCCTACAC-3′
			cyp17a1-R	5′-CCGTATCGTTGCTTCCAGGT-3′

KX420679	*star*—*steroidogenic acute regulatory protein*	111	star-F	5′-AGTACCCTGACCGCCTGTAT-3′
			star-R	5′-TTGTGTCCTGCCCAATCCTC-3′

KX420681	*β-actin*	161	*β*-actin-F	5′-TGACCCTGAAGTAYCCMATC-3′
			*β*-actin-R	5′-CTTCTCTCTGTTRGCYTTGG-3′

KX420682	*gapdh—glyceraldehyde 3-phosphate dehydrogenase*	114	gapdh-F	5′-AGACACCCGCTCNTCHATCT-3′
			gapdh-R	5′-TCCACGACTCTGTTGCTGTA-3′

KX420680	*28S rRNA*—*28S ribosomal RNA*	160	28S-F	5′-TGTTTGTGAATGCAGCCCAA-3′
			28S-R	5′-GACCCCATCCGTTTACCTCT-3′

**Table 2:** The 2^−Δ*C*q^ of each gene. The arithmetic mean of *gapdh* and *β-actin* reference genes was used for normalization. The values are represented by arithmetic means ± SD. One-way ANOVA was used to compare gene expression levels of males versus females for the same organ. NS: not significant.

Organ	*foxl2*			*cyp17a1*			*ar*			*dmrt1*			*sox9*			*star*		
	Mean	±SD	*p* value	Mean	±SD	*p* value	Mean	±SD	*p* value	Mean	±SD	*p* value	Mean	±SD	*p* value	Mean	±SD	*p* value
White muscle ♂	4.75E-02	2.17E-02	NS				1.35E-02	6.46E-03		1.35E-02	7.89E-03	NS	1.53E-03	8.10E-04	NS			NS
White muscle ♀	6.94E-02	3.32E-02					3.58E-02	2.19E-02		3.58E-02	1.10E-02		3.92E-03	2.10E-03				
Liver ♂	3.35E-02	7.33E-03	NS				5.48E-02	2.68E-03		5.48E-02	1.25E-03	NS	8.67E-03	1.27E-03	NS	7.70E-04	1.62E-04	NS
Liver ♀	4.27E-02	2.83E-02					6.93E-02	5.22E-02		6.93E-02	2.39E-02		1.88E-02	7.82E-03		9.23E-04	2.09E-04	
Body kidney ♂	3.38E-03	8.74E-04	NS				8.90E-03	1.02E-03		8,90E-03	1.60E-04	NS	4.24E-03	9.62E-04	NS	2.18E-04	5.30E-05	NS
Body kidney ♀	2.10E-03	1.20E-03					7.64E-03	9.64E-04		7.64E-03	4.17E-04		3.64E-03	8.22E-04		7.80E-03	1.53E-02	
Testicle	6.59E-03	3.44E-03	NS	2.67E-01	7.10E-02	*p* ≤ 0.0001	1.48E-01	9.26E-02	*p* ≤ 0.01	1.48E-01	2.03E-01	*p* ≤ 0.01	1.76E-01	8.62E-03	*p* ≤ 0.0001	1.28E-02	4.17E-04	NS
Ovary	2.99E-02	2.05E-02		3.29E-04	2.62E-04		5.40E-03	2.25E-03		5.40E-03	7.03E-03		2.02E-02	1.35E-02		1.42E-03	8.71E-04	
Anal fin ♂	2.18E-02	8.82E-03	NS	2.38E-04	2.72E-05	NS	1.81E-02	1.18E-03	NS	1.81E-02	4.24E-03	NS	3.74E-02	3.06E-03	NS	2.39E-04	1.42E-04	NS
Anal fin ♀	2.77E-02	2.72E-02		4.69E-04	1.97E-04		1.72E-02	4.27E-03		1.72E-02	1.23E-03		3.86E-02	2.91E-03		8.61E-04	7.13E-04	
